# Characteristics of Epigenetic Clocks Across Blood and Brain Tissue in Older Women and Men

**DOI:** 10.3389/fnins.2020.555307

**Published:** 2021-01-07

**Authors:** Francine Grodstein, Bernardo Lemos, Lei Yu, Artemis Iatrou, Philip L. De Jager, David A. Bennett

**Affiliations:** ^1^Rush Alzheimer’s Disease Center, Rush University Medical Center, Chicago, IL, United States; ^2^Department of Internal Medicine, Rush University Medical Center, Chicago, IL, United States; ^3^Department of Environmental Health, Harvard TH Chan School of Public Health, Boston, MA, United States; ^4^Department of Neurological Sciences, Rush University Medical Center, Chicago, IL, United States; ^5^Department of Neurology and Taub Institute for Research on Alzheimer’s Disease and the Aging Brain, Center for Translational and Computational Neuroimmunology, Columbia University Irving Medical Center, New York, NY, United States

**Keywords:** dementia, epigenetics, aging, neurology, biomarkers

## Abstract

Epigenetic clocks are among the most promising biomarkers of aging. It is particularly important to establish biomarkers of brain aging to better understand neurodegenerative diseases. To advance application of epigenetic clocks—which were largely created with DNA methylation levels in blood samples—for use in brain, we need clearer evaluation of epigenetic clock behavior in brain, including direct comparisons of brain specimens with blood, a more accessible tissue for research. We leveraged data from the Religious Orders Study and Rush Memory and Aging Project to examine three established epigenetic clocks (Horvath, Hannum, PhenoAge clocks) and a newer clock, trained in cortical tissue. We calculated each clock in three different specimens: (1) antemortem CD4+ cells derived from blood (*n* = 41); (2) postmortem dorsolateral prefrontal cortex (DLPFC, *n* = 730); and (3) postmortem posterior cingulate cortex (PCC, *n* = 186), among older women and men, age 66–108 years at death. Across all clocks, epigenetic age calculated from blood and brain specimens was generally lower than chronologic age, although differences were smallest for the Cortical clock when calculated in the brain specimens. Nonetheless, we found that Pearson correlations of epigenetic to chronologic ages in brain specimens were generally reasonable for all clocks; correlations for the Horvath, Hannum, and PhenoAge clocks largely ranged from 0.5 to 0.7 (all *p* < 0.0001). The Cortical clock outperformed the other clocks, reaching a correlation of 0.83 in the DLFPC (*p* < 0.0001) for epigenetic vs. chronologic age. Nonetheless, epigenetic age was quite modestly correlated across blood and DLPFC in 41 participants with paired samples [Pearson r from 0.21 (*p* = 0.2) to 0.32 (*p* = 0.05)], indicating that broader research in neurodegeneration may benefit from clocks using CpG sites better conserved across blood and brain. Finally, in analyses stratified by sex, by pathologic diagnosis of Alzheimer disease, and by clinical diagnosis of Alzheimer dementia, correlations of epigenetic to chronologic age remained consistently high across all groups. Future research in brain aging will benefit from epigenetic clocks constructed in brain specimens, including exploration of any advantages of focusing on CpG sites conserved across brain and other tissue types.

## Introduction

Chronologic age is the strongest risk factor for many chronic diseases; however, disease risk is heterogeneous within age groups, likely due, in part, to variation in “biologic age.” Substantial research has explored biomarkers of aging (Jylhava et al., 2017), which are critical tools for predicting disease risk, assessing mechanisms underlying aging processes, and developing interventions to delay aging-associated declines in health. Epigenetic modifications are a hallmark of aging, and epigenetic clocks are among the most promising biomarkers of aging to date (Jylhava et al., 2017). However, the majority of research establishing the relevance of epigenetic clocks has largely focused on their relations with overall longevity (Fransquet et al., 2019). In the US, heart disease and cancer mortality, primary causes of death, have decreased (Siegel et al., 2018; Shah et al., 2019) while deaths due to Alzheimer dementia and related dementias have increased 145% over the last 20 years (Alzheimer’s Association, 2019); other neurodegenerative diseases are increasing as well (e.g., Parkinson Disease; Marras et al., 2018). Thus, establishing effective biomarkers of brain aging is particularly important for improving public health in the coming decades, and eventually reducing neurodegenerative diseases of aging.

Epigenetic dysregulation has been clearly implicated in brain aging and neurologic diseases (Klein et al., 2016). In initial evidence, several epigenetic clocks were well-correlated with chronologic age when measured in brain tissue (Lu et al., 2017), and in limited existing research, the Religious Orders Study (ROS) and the Rush Memory and Aging Project (MAP) reported that some epigenetic clocks assessed in brain tissue appear modestly associated with neurodegenerative pathology (Levine et al., 2015, 2018). In a small number of studies, epigenetic age measured in blood has also been related to clinical neurologic outcomes (Marioni et al., 2015; Horvath et al., 2016; Chuang et al., 2017; Raina et al., 2017). However, clearer understanding of epigenetic clock behavior in brain is needed to advance applications of these clocks for brain health.

Specifically, growing research indicates that epigenetic clocks, most of which were built with data across the lifespan and relatively few in the oldest age ranges, may become less accurate at the advanced ages at which many neurodegenerative diseases manifest (Armstrong et al., 2017; El Khoury et al., 2019; Shireby et al., 2020). In particular, at the oldest ages—ranging up to supercentenarians (Horvath et al., 2015)—epigenetic age estimates for many of the clocks appear consistently lower than chronologic age. Thus, additional examination of clock behavior at more extreme older ages is needed, especially if the trajectory of biological age may not be linear in advanced age. Further, direct comparisons are needed of epigenetic clock behavior in brain specimens compared to more accessible tissue (e.g., blood). Initial evidence, including in ROS and MAP, has suggested that DNA methylation (DNAm) states do not appear correlated in blood vs. brain specimens (Lunnon et al., 2014; Yu et al., 2016). Nonetheless, this work has examined hundreds of thousands of CpG sites simultaneously, and may not apply to the more focused signatures provided by epigenetic clocks.

Thus, we leveraged the data from the Religious Orders Study and the Rush Memory and Aging Project to examine inter-relations of four different epigenetic clocks measured in three different specimens: (1) antemortem CD4^+^ cells derived from blood (two measures, on average 7.5 years apart); (2) postmortem dorsolateral prefrontal cortex (DLPFC); and (3) postmortem posterior cingulate cortex (PCC), among older women and men, age 66–108 years at death. We chose to focus here on a range of different epigenetic clocks: the Horvath clock, developed across multiple tissues (Horvath, 2013); the Hannum clock, trained in blood samples (Hannum et al., 2013); the PhenoAge clock, developed with biomarkers of aging as the dependent variable rather than age (Levine et al., 2018); and a new clock trained in cortical tissue (Cortical clock; Shireby et al., 2020).

## Materials and Methods

### Study Populations

The Religious Orders Study (Bennett et al., 2018) was initiated in 1994, and includes older priests, nuns and brothers from across the United States, free of known dementia at the time of enrollment. Participants agreed to annual neurological exams, neuropsychological testing, and blood draw, and signed an informed consent and Anatomic Gift Act to donate their brains at death. Over 1,468 participants completed a baseline evaluation. The follow-up rate and autopsies exceed 90%. The Rush Memory and Aging Project (Bennett et al., 2018) was established in 1997 with virtually identical design and data collection, and includes older men and women from across the Chicago metropolitan area, without known dementia at enrollment; over 2,170 participants completed a baseline evaluation to date. The follow-up rate exceeds 90% and the autopsy rate exceeds 80%. ROSMAP data can be requested at www.radc.rush.edu.

For the work described here, we leveraged DNA methylation profiling previously completed in stored peripheral blood samples collected from participants at cohort baseline and again proximate to death, as well as from frozen DLPFC and PCC tissue. The average postmortem interval was approximately 9 h.

### Assessment of DNA Methylation States and Epigenetic Clocks

First, DNAm was profiled in a set of 41 matched blood samples and DLPFC specimens. The blood DNAm was profiled in CD4^+^ T cells isolated from frozen peripheral blood mononuclear cells (PBMCs). For the original research, we had been interested in CD4^+^ lymphocytes because they represent a single cell type related to immune function. As previously described (De Jager et al., 2014), the PBMCs were washed with RPMI1640 medium to remove Dimethyl sulfoxide (DMSO) exposure. CD4^+^ T-cells were isolated using magnetic-activated cell sorting (MACS) and reached the purity of at least 95% as assessed by flow cytometry. Blood DNA isolation was performed using AllPrep DNA/RNA Micro kit, according to manufacturer’s instructions.

In the DLPFC, 100 mg frozen sections were thawed on ice, with the gray matter dissected from the white matter. The Qiagen QIAamp DNA mini protocol was used for DNA isolation, as previously published (De Jager et al., 2014). In the blood and DLPFC, DNA methylation profiles were generated using the Illumina Infinium HumanMethylation450 platform. Details on the processing and quality control pipelines have been previously described in detail (De Jager et al., 2014; Yu et al., 2016). After processing, data on 420,132 CpG sites were retained across the 22 autosomes. At each site, DNAm level was presented as a beta value, that is, the ratio of the methylated probe intensity to the sum of methylated and unmethylated probe intensities. The values ranged from 0 to 1, where a larger value indicates higher methylation.

In more recent work with the larger set of DLPFC specimens (*n* = 730) and the PCC (*n* = 186), processing methods were updated. Thus, the data presented for the blood and matched DLPFC maintain the original pipelines, while the data presented for the full set of 730 DLPFC and the 186 PCC utilize the updated pipelines. For the PCC, we also updated to the Infinium MethylationEPIC array. For the full set of DLPFC and PCC, the raw signal intensities were imported into the R statistical environment with functions from the methylumi package and further processed with the wateRmelon package. The pipeline for quality control was generally consistent for the HumanMethylation450 and the MethylationEPIC arrays. Initial quality control assessment was performed using functions in the methylumi package to exclude samples with inefficient bisulfite conversion (<90%) as well as outliers. Further preprocessing was conducted using the wateRmelon package by applying a p-filter. Probes having more than 1% of samples with a detection *p*-value greater than 0.05 and a beadcount lower than 3 in more than 5% of samples were excluded. Finally, the filtered data were normalized with “dasen.” Non-CpG SNP (Single Nucleotide Polymorphism) probes, probes that had been reported to contain common (MAF > 5%) SNPs in the CG or single base extension position, or probes that were non-specific or mismapped, were flagged and disregarded in the evaluation of our results. The resulting datasets for analysis here consisted of 730 samples with 423,841 probes each for the DLPFC, and 186 samples with 810,015 probes each for the PCC. Adequate information for probes relevant to the four clocks was available after all processing.

We used open source software at https://dnamage.genetics.ucla.edu/home_ to calculate three epigenetic clocks in the blood samples, DLPFC, and PCC: Horvath clock (Horvath, 2013), Hannum clock (Hannum et al., 2013), and PhenoAge clock (Levine et al., 2018). The Horvath clock is a pan-tissue clock, originally constructed utilizing CpG sites across 51 human cell types and tissues. The clock was designed by regressing DNAm states on chronologic age and using elastic net regression to identify a prediction model; it combines information from 353 CpG sites to calculate epigenetic age. The Hannum clock was developed similarly, by regressing DNAm states on chronologic age, although only in peripheral blood samples, and includes 71 CpG sites. The PhenoAge clock was created in blood samples, but regressed DNAm states on clinical biomarkers rather than on chronologic age; it incorporate 513 CpG sites. The Cortical clock was calculated using publically available code provided by the authors^1^.

### Populations for Analysis

For examining epigenetic clocks in the CD4^+^ cells, we leveraged information from 41 ROS or MAP participants, who also had archived DLPFC. The 41 participants were identified from a subset of those who provided annual blood, and had samples from baseline and proximate to death (mean = 7.5 years of follow-up). For further examination focused in the DLPFC, we used 730 specimens that were part of previous research on DNAm and neurodegeneration; analyses of the Cortical clock in DLPFC excluded 88 specimens, which had been part of the original training set for the Cortical clock. Finally, for the PCC, 186 samples were available at the time we conducted these analyses. For examining correlations across tissues, we examined 41 participants with both blood samples and DLPFC, as well as 90 women and men who had information on epigenetic clocks in both DLPFC and PCC. No participant had DNAm profiles across all three specimens.

### Statistical Analysis

First, we examined the Pearson correlations of each epigenetic clock, in each specimen type, to chronologic age at specimen collection. In addition, since previous research (Armstrong et al., 2017; El Khoury et al., 2019; Shireby et al., 2020) has noted that epigenetic clock age often underestimates chronologic age in older age groups, we created quintiles of chronologic age, and then examined the difference of epigenetic and chronologic age separately within each quintile, for each clock. These age group analyses were conducted in the DLPFC and PCC specimens, due to the larger sample sizes available.

Next, we considered the correlations of epigenetic clock ages across the two timepoints within the CD4^+^ cells, as well as clock ages across the 41 matched CD4^+^ and DLPFC, and across the 90 matched DLPFC and PCC. To understand how the various clocks relate to each other, within each specimen, we also compared epigenetic clock ages across the four clocks (e.g., correlation of the Horvath to Hannum clock within baseline blood samples). To help evaluate the difference between epigenetic and chronologic age, we also calculated the residuals from regressing epigenetic age on chronologic age for each specimen type (which has been termed “age acceleration”). Analyses separately examined both epigenetic age and epigenetic age acceleration.

Finally, we considered how key factors such as sex, Alzheimer disease (AD) neuropathology, and clinical health status may affect clock behavior. We conducted analyses of the correlation of epigenetic to chronologic age separately according to: sex (male/female); pathologic diagnosis of Alzheimer disease (yes/no); and clinical diagnosis of Alzheimer dementia (yes/no). Ascertainment of and pathologic AD was identified using the NIA/Reagan criteria, and clinical Alzheimer dementia was assessed by experienced clinicians, using cognitive and clinical data, as previously described (McKhann et al., 1984; National Institute on Aging Reagan Institute Working Group on Diagnostic Criteria for the Neuropathological Assessment of Alzheimer’s Disease, 1997; Bennett et al., 2006). These subgroup analyses included only DLPFC and PCC, due to the larger sample sizes available in these specimens.

## Results

Chronologic age was approximately 81 years (standard deviation, SD, 6.2) at the baseline blood collection, 89 years (SD 4.7) at the second blood collection proximate to death, and nearly 90 years (SD 4.9) in the matched postmortem DLPFC (*n* = 41). For the full set of brain specimens, mean age at death was 88.0 (SD 6.7) in those with DLPFC (*n* = 730) and 90.0 (SD 6.0) years in those with PCC (*n* = 186) (Table 1). Approximately 2/3 of participants were female. Clinical diagnosis of Alzheimer dementia as of death was common, ranging from approximately half of participants with a blood sample, to approximately one-third of those with PCC specimens. Pathologic diagnosis of AD was highly prevalent—60% of participants with a blood sample and over 70% of those with brain specimens.

**TABLE 1:** Characteristics of participants: Religious Orders Study and Memory and Aging Project.

	**Populations for analysis, according to specimen types^*a*^**				
	**Baseline blood**	**Blood proximate to death**	**DLPFC with matched blood**	**All DLPFC^*b*^**	**PCC**
**N**	41	41	41	730	186
**Chronologic Age (mean, SD)**	81.2 (6.2)	88.7 (4.7)	89.6 (4.9)	88.0 (6.7)	90.0 (6.0)
**Female (%)**	66%	66%	66%	64%	66%
**Clinical Alzheimer Dementia**	0	54%	54%	42%	33%
**Pathologic Alzheimer Disease**	n/a	73%	73%	60%	60%
**Epigenetic Age (Mean, SD, range)**					
** Horvath clock**	64.6 (8.4) Range:52.4,87.8	67.6 (7.2) Range:49.7,90.4	69.3 (5.3) Range: 59.6,82.6	79.7 (6.3) Range:60.8,103.5	71.3 (3.9) Range:56.4,82.6
** Hannum clock**	70.1 (6.8) Range:57.2,88.3	73.6 (6.5) Range:54.4,87.8	51.4 (3.3) Range:43.8,59.5	57.0 (3.2) Range:44.8,66.4	59.1 (2.5) Range:49.3,64.4
** PhenoAge clock**	59.8 (8.8) Range:39.0,87.5	63.8 (8.1) Range:43.7,80.9	4.5 (5.4) Range:-6.4,23.4	1.6 (5.8) Range:-16.9,24.7	12.1 (4.3) Range:1.5,39.7
** Cortical clock**	50.3 (8.1) Range:27.9,65.4	53.4 (7.9) Range:39.2,71.3	79.5 (5.6) Range:70.6,94.8	87.3 (5.6) Range:65.0,102	95.9 (5.2) Range:79.1,124

*^*a*^DLPFC, dorsolateral prefrontal cortex; PCC, posterior cingulate cortex. ^*b*^For analyses of the Cortical clock in the set of all DLPFC, we excluded 88 specimens which had been part of the original training set for this clock, thus, n = 642 for the Cortical clock.*

Across virtually all specimen types, mean epigenetic age was lower than mean chronologic age (Table 1). In the blood samples, the PhenoAge and Cortical clocks produced the largest differences between mean chronologic and epigenetic ages. Specifically, mean PhenoAge was 59.8 (SD 8.8) in the baseline blood samples and 63.8 (SD 8.1) in the blood proximate to death; Cortical age was 50.3 (SD 8.1) in the baseline blood and 53.4 (SD 7.9) in the blood proximate to death. Interestingly, in the blood samples, mean Hannum clock age was closest to chronologic age (baseline blood collection: mean Hannum age = 70.1, SD 6.8; blood proximate to death: mean = 73.6, SD 6.5), likely because the Hannum clock was originally constructed in peripheral blood. Finally, we could consider change in epigenetic clock age over time within the two paired blood samples; as expected, the average epigenetic age was greater in the second than the first blood sample for all four clocks. Nonetheless, the difference in mean epigenetic age over the two timepoints was approximately 3–5 years across all four clocks, while the corresponding change in chronologic age was 7.5 years.

In the brain specimens (Table 1), mean Cortical age was closest to mean chronologic age (DLPFC: mean Cortical age = 86.5, SD 6.0; PCC: mean = 95.9, SD 5.2). Further, mean Cortical age in the PCC was greater than chronologic age, while clock age was lower than chronologic age for all the other clocks. In particular, PhenoAge was substantially younger than chronologic age in the brain specimens, with a mean of 1.6 (SD 5.8) in DLPFC and 12.1 (SD 4.3) in PCC.

**FIGURE 1:**
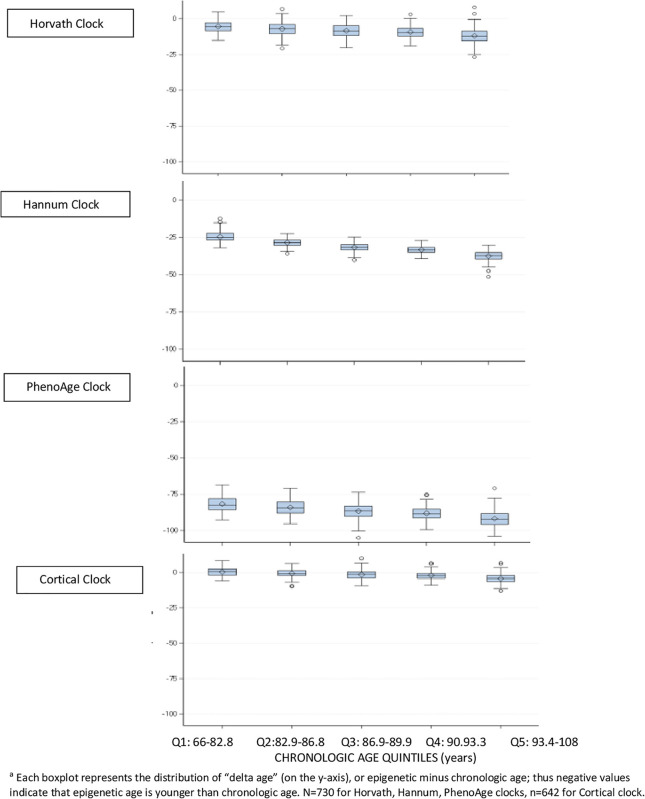
Difference between epigenetic clock age and chronologic age^*a*^, within quintiles of chronologic age, in dorsolateral prefrontal cortex.

To more closely examine differences between epigenetic and chronologic ages in the brain specimens, we divided the population into quintiles of age, and constructed boxplots of “delta age” by subtracting chronologic from epigenetic age (Figure 1); thus, negative values of delta age indicate that epigenetic age is younger than chronologic age. In DLPFC, for the Horvath, Hannum, and PhenoAge clocks, within every quintile of age, the median delta age as well as the upper 25th percentile of the distribution were all negative (Figure 1). However, of these three clocks, the smallest delta ages were consistently observed for the Horvath multi-tissue clock. Yet, for the Cortical clock, median delta age was positive (median = 0.7 years) in the youngest quintile of age, and the cutpoints for the upper 25th percentile of the distribution were positive through the youngest three quintiles of age. Thus, Cortical clock was the only one which did not largely underestimate chronologic age at the younger ages in this sample. Nonetheless, across all four clocks, median delta age became larger and more negative with each older age group. For example, in the oldest quintile of age, median delta age was 12.1 years, 37.3, 92.3, and 4.2 for the Horvath, Hannum, PhenoAge and Cortical clocks, respectively. That is, all clocks underestimated chronologic age by greater amounts with older age of the participants.

These findings were all generally consistent in the PCC (data not shown), although Cortical age in the PCC was greater than chronologic age in virtually all samples.

**FIGURE 2:**
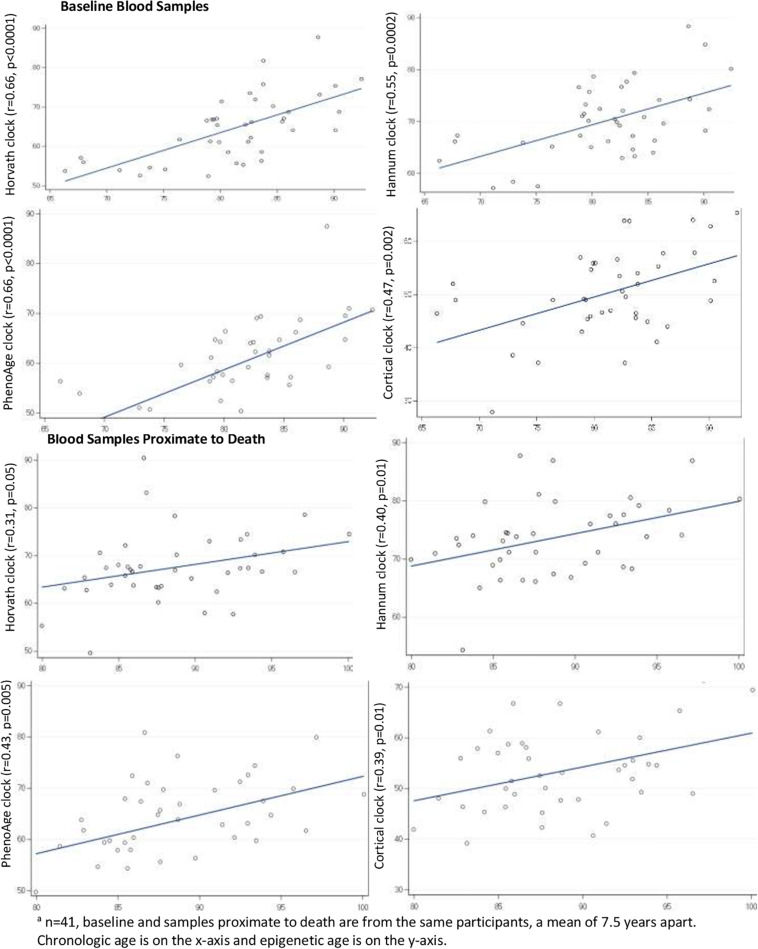
Pearson correlations of epigenetic age to chronologic age in blood samples^*a*^.

Next, we directly examined correlations of clock age to chronologic age within the blood samples (Figure 2), correlations were generally reasonable for the Horvath, Hannum, PhenoAge and Cortical clocks, ranging from a low of 0.31 (Horvath clock in the blood proximate to death) to a high of 0.66 (Horvath and Hannum clocks in the baseline blood). Across the clocks, correlations tended to be lowest in the blood samples proximate to death (range 0.31–0.43), and correlations tended to be lower for the Cortical clock than the others; for example, the correlation of Cortical to chronologic age was 0.47 in the baseline blood (the lowest of all four clocks).

**FIGURE 3:**
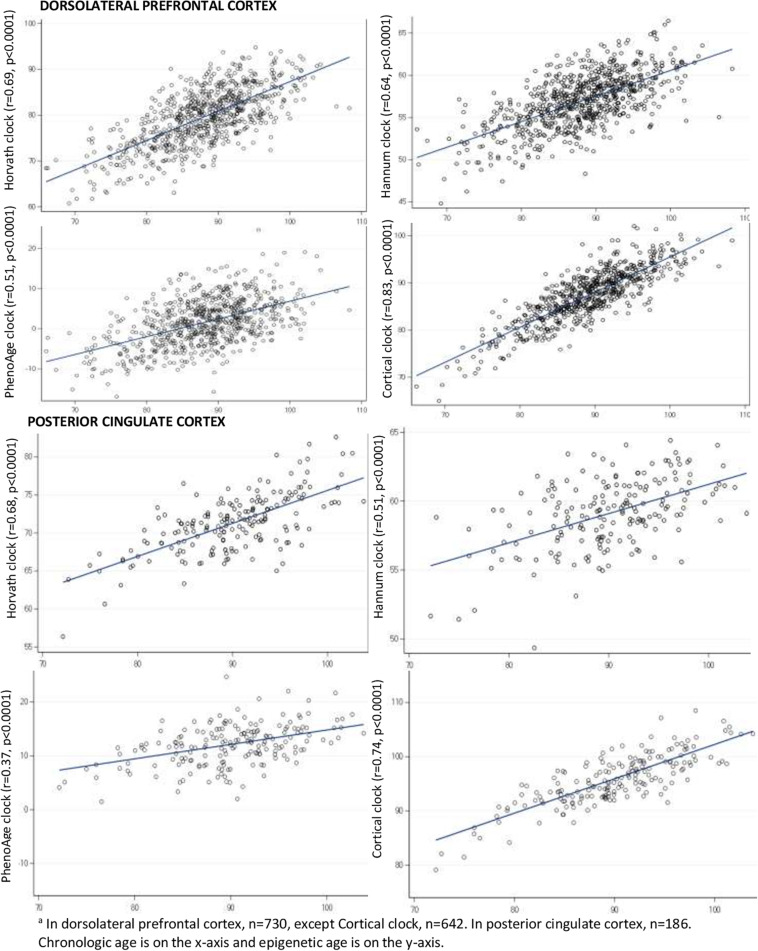
Pearson correlations of epigenetic age to chronologic age in brain specimens^*a*^.

In brain specimens (Figure 3), correlations of epigenetic to chronologic age for the Horvath, Hannum, and PhenoAge clocks largely ranged from approximately 0.5–0.7. The PhenoAge clock consistently performed worst of the four clocks, with a correlation of 0.51 in DLPFC and 0.37 in PCC. The Horvath clock, the only multi-tissue clock, had higher correlations than either the Hannum or PhenoAge clocks in brain specimens. However, the Cortical clock performed best in both the DLPFC and PCC (*r* = 0.83 in DLPFC, *r* = 0.74 in PCC).

For each clock, we also correlated epigenetic ages across paired specimens (Table 2), including the two blood samples over time (*n* = 41), the matched blood and DLPFC specimens (*n* = 41), and the two cortical regions (*n* = 90). For the two blood samples, collected an average of 7.5 years apart, correlations were 0.32 for the PhenoAge clock over two timepoints, 0.42 for the Horvath clock, 0.51 for the Hannum clock, and 0.53 for the Cortical clock. When comparing clock ages in blood vs. brain specimens, we found fairly low correlations of the baseline blood sample or the blood sample proximate to death with the postmortem DLPFC; the Horvath and Hannum clocks tended to have better correlations than the other clocks (e.g., *r* = 0.31 and 0.30, respectively, for baseline blood sample). However, we found the highest correlations across specimen types when we compared epigenetic age across the two cortical regions (Horvath: *r* = 0.61; Hannum *r* = 0.40; PhenoAge *r* = 0.37); this correlation was particularly high for the Cortical clock (*r* = 0.82). In additional analyses to explore whether there may be better correlations when considering the extent of epigenetic age acceleration across specimens than the extent of epigenetic aging, we found that results were generally similar for clock age acceleration (data not shown in table) as for clock age.

**TABLE 2:** Pearson correlations of epigenetic clocks across blood samples and brain regions.

		**Correlation across specimen types, for each epigenetic clock (r, *p*-value)**			
***Sets of biospecimens***	***N***	**Horvath clock**	**Hannum clock**	**PhenoAge clock**	**Cortical clock**
**Baseline blood/blood proximate to death**	41	0.42 (*p* = 0.007)	0.51 (*p* = 0.0007)	0.32 (*p* = 0.03)	0.53 (*p* = 0.0003)
**Baseline blood/DLPFC**	41	0.31 (*p* = 0.05)	0.30 (*p* = 0.05)	0.08 (*p* = 0.6)	0.24 (*p* = 0.1)
**Blood proximate to death/DLPFC**	41	0.32 (*p* = 0.04)	0.31 (*p* = 0.05)	0.21 (*p* = 0.2)	0.19 (*p* = 0.2)
**DLPFC/PCC**	90	0.61 (*p* = 0.0001)	0.40 (*p* = 0.0001)	0.37 (*p* = 0.0003)	0.82 (*p* < 0.0001)

Within each specimen, when we compared the various clocks to each other (Table 3), overall, correlations were 0.65 or greater for over half of the comparisons. The lowest correlations tended to be for the PhenoAge vs. other clocks, perhaps since the PhenoAge clock was the only clock designed to predict biomarkers of aging rather than chronologic age.

**TABLE 3:** Pearson correlations of epigenetic clocks to each other, by specimen type.

		**Correlation of epigenetic clocks to each other (r, *p*-value)**					
***Biospecimen type***	***N***	**Horvath vs. Hannum clock**	**Horvath vs. PhenoAge clock**	**Horvath vs. Cortical clock**	**Hannum vs. PhenoAge clock**	**Hannum vs. Cortical clock**	**PhenoAge vs. Cortical clock**
Baseline blood	41	0.74 (*p* < 0.0001)	0.69 (*p* < 0.0001)	0.67 (*p* < 0.0001)	0.68 (*p* < 0.0001)	0.80 (*p* < 0.0001)	0.58 (*p* < 0.0001)
Blood proximate to death	41	0.59 (*p* < 0.0001)	0.74 (*p* < 0.0001)	0.65 (*p* < 0.0001)	0.70 (*p* < 0.0001)	0.71 (*p* < 0.0001)	0.67 (*p* < 0.0001)
DLPFC	730	0.71 (*p* < 0.0001)	0.57 (*p* < 0.0001)	0.79 (*p* < 0.0001)	0.44 (*p* < 0.0001)	0.71 (*p* < 0.0001)	0.50 (*p* < 0.0001)
PCC	186	0.65 (*p* < 0.0001)	0.44 (*p* < 0.0001)	0.75 (*p* < 0.0001)	0.18 (*p* = 0.01)	0.56 (*p* < 0.0001)	0.46 (*p* < 0.0001)

**FIGURE 4:**
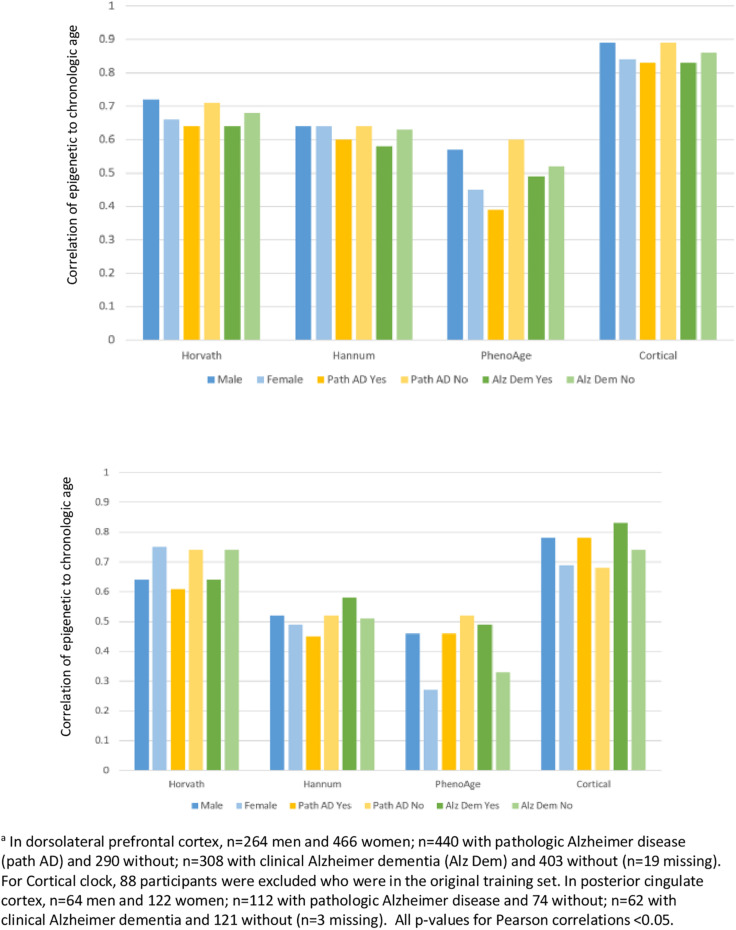
Pearson correlations of epigenetic age to chronologic age, according to characteristics of participants^*a*^.

Finally, we examined how clock performance may differ in men vs. women, or in those with differing health status (Figure 4), in the DLPFC and PCC specimens. Most importantly, the correlations of epigenetic to clock age remained similar across men and women, those with and without pathologic diagnosis of AD, and those with and without clinical diagnosis of dementia. However, there were suggestions of somewhat higher correlations of epigenetic to chronologic age in men than in women, and somewhat lower correlations in those with pathologic AD than without pathologic AD; for example, the correlation of Cortical age to chronologic age in DLFPC was 0.78 among men and 0.69 among women, and was 0.78 in those without pathologic AD compared to 0.68 in those with pathologic AD.

## Discussion

In this investigation of characteristics of epigenetic clocks across blood and brain specimens in older adults, we confirmed previous reports (Armstrong et al., 2017; El Khoury et al., 2019; Shireby et al., 2020), that epigenetic age was generally lower than chronologic age, across specimen types. Specifically, for the Horvath, Hannum, and PhenoAge clocks, median epigenetic age was lower than chronologic age from the youngest through the oldest quintiles of age in our sample. By contrast, as may be expected, the Cortical clock demonstrated the smallest differences between epigenetic and chronologic age in DLPFC and in PCC. Indeed, the Cortical clock was not only designed in brain tissue, but the training set included much larger samples of older participants than the other clocks, which certainly further enhances its accuracy in estimating brain aging. We also extended published findings (Lu et al., 2017; Shireby et al., 2020), using varying clocks than previously examined, that correlations of chronologic to epigenetic ages in brain specimens (i.e., DLPFC and PCC) were generally reasonable for blood-based and multi-tissue clocks. In our specimens, these correlations largely ranged from 0.5 to 0.7 for the Horvath, Hannum and PhenoAge clocks—despite none being designed expressly in brain tissue. We further confirmed a previous report (Shireby et al., 2020) that the new Cortical clock—the first designed in post-mortem brain tissue—performed substantially better in brain specimens than the other clocks, with a correlation of epigenetic vs. chronologic age over 0.8 in our DLPFC. Thus, our findings both provide broad support for the value of epigenetic clocks in research on brain aging, as well as specific support for “bespoke” clocks (Bell et al., 2019) designed in target tissues of interest. Finally, however, epigenetic age was only quite modestly correlated across paired blood and DLPFC, indicating that broader research in biologic aging and neurodegeneration may benefit from epigenetic clocks focused in CpG sites better conserved across blood and brain specimens.

Numerous large-scale studies have reported good correlations of chronologic to epigenetic age in peripheral blood, similar to our findings (Chen et al., 2016). However, less is known regarding epigenetic aging specifically in blood samples proximate to death. In our study of 41 blood samples collected a mean of 0.9 years prior to death, we found that the correlation of chronologic to epigenetic age appeared worse than in the baseline samples collected years earlier. Specifically, these correlations ranged from 0.3 to 0.4. At the same time, from the baseline to the final blood collection, the correlations of epigenetic ages over these 7 years in our study (0.32–0.53) were similar to those reported in other studies with repeated measures of blood DNAm, including studies which did not focus on blood collected proximate to death (Marioni et al., 2019). For example, over approximately 7 years, in two other studies (Marioni et al., 2019) (*n* = 172 and 175), they found correlations of 0.33–0.64 for the Horvath and for the Hannum clock over time, suggesting that DNAm levels in our study changed in at least somewhat expected ways. Ultimately, our sample size for the peripheral blood was not large, thus it is difficult to ascertain a cause of the observed lower correlations in the blood samples proximate to death. However, our findings indicate that further specific investigations of peripheral blood proximate to death may provide new understanding of DNA methylation in health and mortality.

Considerably less is known regarding epigenetic age in brain tissue. In one publication reporting data only on the Horvath clock, using seven smaller cohorts (*n* = 37–302, including a subset of the ROS and MAP DLPFC here), Lu et al. (2017) observed correlations of chronologic age to clock age ranging from 0.61 to 0.99 within 6 brain regions. In more recent research of PhenoAge, Levine et al. (2018) reported correlations with chronologic age of 0.51–0.92 across varying brain regions (including DLPFC in ROSMAP). Thus, both of these publications found largely similar correlations as we report here, supporting the relevance of epigenetic clocks to brain aging. Moreover, we found especially high correlations of Cortical age to chronologic age in the DLPFC and PCC—and excellent correlation of Cortical age across the DLPFC and PCC. Further, in limited existing clinical research in brain tissue, the Horvath clock in ROSMAP DLPFC was related to some neuropathologic measures, with significant correlations of DNAm age to amyloid load, neuritic plaques, and diffuse plaques, but not to tangles, pathologic AD or to clinical measures of cognitive function (Levine et al., 2015). Interestingly, although we found that the PhenoAge clock had the lowest correlation with chronologic age in brain tissue, initial analyses by Levine et al. (2018) suggested that PhenoAge in the ROSMAP DLPFC was also significantly related to amyloid load, to neurofibrillary tangles, as well as to pathologic AD diagnosis. It will clearly be important in future research to extensively explore each of the clocks, assessed in brain tissue, in association with neuropathologic and clinical neurologic outcomes. In particular, while the Cortical clock here was best correlated to chronologic age in the brain tissue, and had the smallest absolute difference between epigenetic and chronologic age, it will be interesting and important to evaluate whether this also translates into higher predictive ability for neuropathologic and clinical neurologic outcomes above and beyond the effect of chronologic age.

Notably, we also found that correlations of epigenetic age in blood vs. brain samples were low, largely 0.3 or lower across the clocks. While research focused in brain specimens remains central to understanding neurodegeneration, epigenetic clocks that could allow direct translation across brain and blood, will have the greatest research potential, given the accessibility of blood samples. Future development of epigenetic clocks for translational research in neurodegenerative diseases might benefit from focusing on CpG sites which are conserved across blood and brain tissue, as an approach to explore for improved relevance in both tissue types.

Finally, when we specifically examined brain specimens from participants according to sex or to health profiles, we found that the clocks consistently performed well in males and females, and in those with or without either pathologic diagnosis of AD or clinical diagnosis of dementia. This is reassuring evidence that underlying characteristics of participants do not appear to have material influence on basic functioning of the epigenetic clocks. Interestingly, the Cortical clock is the only clock trained in a sample which excluded Alzheimer disease cases, due to concerns that underlying disease could potentially influence results (Shireby et al., 2020). However, in comparison to the other clocks, the Cortical clock did not demonstrate correlations with chronologic age that were consistently better or worse in our participants with vs. without pathologic AD, or clinical dementia; this may further support the ability of epigenetic clocks to estimate broad biologic aging across and within specific underlying disease states.

Strengths of our study include the unique availability of paired blood samples and brain specimens, enabling investigation of epigenetic clocks across these tissues. Further, our large sample of older persons permitted detailed examination of epigenetic clocks from brain tissue at older ages. There are limitations as well. The proportion of neurons and other cell types in the gray matter may confound DNAm states, which we did not consider here; however, in previous analyses in ROSMAP DLPFC, control for neuron count when broadly examining DNAm profiles or specifically examining epigenetic clocks, did not meaningfully change results (Horvath et al., 2015; Levine et al., 2018). The blood samples were limited to CD4^+^ cells, whereas prior studies examined clocks built from a wide variety of cell types (Fransquet et al., 2019); however, our findings largely mirror those of studies using varying blood cells. Nonetheless, it is possible that specific findings here (e.g., the generally low correlations between clocks in blood vs. brain tissue), might be different in other blood cell types, a topic meriting additional research. Further, the sample size of blood specimens, including matched blood and brain specimens, was small, limiting our analyses and interpretations. Finally, we did not consider how the epigenetic clocks may predict the span of neuropathologic or clinical neurologic outcomes. Instead, we chose to focus here on extensive consideration of basic characteristics of epigenetic clocks in blood and brain specimens—a crucial step prior to broader neurologic research. In future investigations, we will more directly address relations of these clocks to brain pathology and to cognition.

## Data Availability Statement

Publicly available datasets were analyzed in this study. The ROSMAP datasets analyzed here can be requested at https://www.radc.rush.edu/.

## Ethics Statement

The studies involving human participants were reviewed and approved by the Rush University Medical Center IRB. The patients/participants provided their written informed consent to participate in this study.

## Author Contributions

FG and BL drafted the manuscript and contributed to conception, design, and analysis and interpretation of data. LY revised the manuscript, and contributed to conception and design of the research, and data acquisition, analysis, and interpretation. AI revised the manuscript and contributed to data acquisition and interpretation. PD revised the manuscript, and contributed to conception and design of the research, and data acquisition and interpretation. DB revised the manuscript, and contributed to conception and design of the research, and data acquisition, analysis and interpretation. All authors contributed to the article and approved the submitted version.

## Conflict of Interest

The authors declare that the research was conducted in the absence of any commercial or financial relationships that could be construed as a potential conflict of interest.

## Footnotes

1 https://github.com/gemmashireby/CorticalClock
